# Local Destruction of Tumors and Systemic Immune Effects

**DOI:** 10.3389/fonc.2021.708810

**Published:** 2021-07-08

**Authors:** Karl-Göran Tranberg

**Affiliations:** Department of Surgery, Lund University, Lund, Sweden

**Keywords:** local treatment, immunotherapy, abscopal, tumor antigen, tumor cell death, tissue perfusion, presentation, trafficking

## Abstract

Current immune-based therapies signify a major advancement in cancer therapy; yet, they are not effective in the majority of patients. Physically based local destruction techniques have been shown to induce immunologic effects and are increasingly used in order to improve the outcome of immunotherapies. The various local destruction methods have different modes of action and there is considerable variation between the different techniques with respect to the ability and frequency to create a systemic anti-tumor immunologic effect. Since the abscopal effect is considered to be the best indicator of a relevant immunologic effect, the present review focused on the tissue changes associated with this effect in order to find determinants for a strong immunologic response, both when local destruction is used alone and combined with immunotherapy. In addition to the T cell-inflammation that was induced by all methods, the analysis indicated that it was important for an optimal outcome that the released antigens were not destroyed, tumor cell death was necrotic and tumor tissue perfusion was at least partially preserved allowing for antigen presentation, immune cell trafficking and reduction of hypoxia. Local treatment with controlled low level hyperthermia met these requisites and was especially prone to result in abscopal immune activity on its own.

## Introduction

Modern immunotherapies, especially therapy with checkpoint blockers (CPBs), have revolutionized cancer treatment. It began in 2011 when CTLA-4 (cytotoxic T-lymphocyte-associated protein 4) blocking antibody was approved by the Food and Drug Administration (FDA) for treatment of patients with metastatic melanoma. Programmed cell death 1 (PD-1) checkpoint inhibitors were approved by FDA for the same disease three years later. Today, CPBs have shown clinical efficacy in randomized controlled trials for a number of solid malignancies.

A decade of clinical experience has shown, however, that most patients do not respond to CPBs in a satisfactory way (1–3), pointing to the need for new or complementary approaches. It would also be beneficial to find ways to reduce cost and the risk for severe, occasionally life-threatening side-effects of the treatment. Addition of immunostimulating local therapy may allow lowering of antibody doses with preserved, or increased, efficacy (3).

Physically based local tumor destruction (LTD) methods have been in clinical use for many years, and numerous studies, mainly preclinical, have demonstrated immunologic effects. There is, however, considerable variation between the different LTDs with respect to the mode of action and frequency and strength of the systemic anti-tumor immunologic effect. Since we do not know why, this review describes the characteristics and outcome for different LTDs with the goal to find properties that are important for a successful immunologic response against malignant disease. The main findings are that presentation of intact tumor antigens, type of cell death and preservation of tumor blood perfusion play important roles for the outcome of LTD.

## Cancer Immunotherapies

Presently, the major cancer immunetherapies^1^ are vaccines, adoptive cell transfer (ACT) and immune checkpoint inhibitors.

### Cancer Vaccines

Therapeutic cancer vaccines have had limited success in the clinical setting A major reason for this seems to be the lack of an immunogenic tumor microenvironment (TME), which is needed for efficient antigen- presenting DCs and efficient anti-tumor T cell activity (4). The bacillus Calmette-Guérin (BCG) vaccine for treatment of early-stage bladder cancer was FDA-approved in 1990. After that, one exogenous anticancer vaccine, sipuleucel-T, for treating asymptomatic or minimally symptomatic metastatic castration-resistant prostate cancer, has been approved (5).

The so called endogenous vaccines work by mobilizing antigens from the patient’s tumor *in situ*. This is accomplished by inducing tumor cell death under conditions that favor the ability of dendritic cells (DCs) to capture, process and present tumor-derived antigens. An advantage of endogenous vaccines is that they may be able to elicit the presentation of a large number of antigens, including tumor-specific neoantigens. One example of endogenous vaccination is oncolytic virus immunotherapy. A modified herpes simplex virus type 1 (HSV-1), engineered to secrete granulocyte-macrophage colony-stimulating factor (GM-CSF), was approved by FDA in 2015 for treatment of advanced melanoma (6).

### Adoptive Cell Transfer

Most adoptive cell transfer strategies use transfer of tumor-infiltrating lymphocytes (TILs) or genetically engineered T cells bearing chimeric antigen receptors (CARs).

The majority of studies with TIL therapy have been in advanced melanoma. In this disease, Rosenberg and coworkers accomplished complete regression in 20/93 patients (22%), with 19 patients not having relapse within 5-10 years (7). The combined experience at the NCI (Bethesda, Maryland) shows an overall response rate (OR) of 55% in 194 patients, and similar results have been obtained at other centers (7).

CARs are constructs that combine the antigen-recognition properties of antibodies with T cell lytic functions. Since CAR receptors are designed to target surface molecules on tumor cells, there is no need for tumor cells to have a working antigen-processing machinery and to be able to express antigen through MHC class I or II (8). FDA has recently approved CAR T cell therapy for some forms of aggressive B cell malignancies.

ACTs face manufacturing challenges and the competition from simpler and cheaper treatments. Their strength is the potential for long-term durable responses and cures.

### Immune Checkpoint Blockers

Immune checkpoint blockers are monoclonal antibodies that inhibit receptors of T lymphocytes that block T cell mediated anti-tumor immunity when the T cell is activated (9, 10). Increased levels of CTLA-4 are displayed on the cell surface, where it binds to members of the B7 family expressed by dendritic cells (DCs) and other antigen-presenting cells (APCs). CTLA-4 interferes with early T cell activation, and anti-CTLA-4 leads to increased activation and proliferation of T cells in lymphoid organs and tumor tissues. CTLA-4 is present also on regulatory T cells (T_reg_ cells) and at least part of the immune suppressive function of T_reg_ is thought to be blocked by anti-CTLA-4.

PD-1 interferes with signaling mediated by the T cell antigen receptor. PD-1 is expressed particularly on activated T cells during the activation phase and regulates the immune response at a later stage during the peripheral tissue infiltration by effector T cells. Cancer cells and tumor-infiltrating immune cells may express PD-L1 (and occasionally PD-L2). PD-1 is also expressed on T_reg_ cells, and it has been shown that anti-PD-1 can decrease T_reg_ levels within tumor tissue (11).

The effect of anti-PD-1 or anti-PD-L1 (PD-L1: programmed death-ligand 1), sometimes combined with anti-CTLA-4, has been investigated in most solid malignancies and has gained FDA approval for use in, for instance, melanoma, non-small cell lung cancer (NSCLC), urothelial cancer, Hodgkin lymphoma and high microsatellite instability cancer. Pancreatic ductal adenocarcinoma, prostate cancer and microsatellite stable colorectal cancer appear relatively unresponsive to immune checkpoint blockade alone (12). Overall, success has been stronger in malignant melanoma than in other solid tumors (1, 2). In a recently published, randomized study, patients with previously untreated advanced melanoma received anti-CTLA-4 alone, anti-PD-1 alone or anti-CTLA-4 plus anti-PD-1 (13). Median progression-free survival was 11.5 months in the combination group, 6.9 months in the anti-PD-1 group and 2.9 months in the anti-CTLA-4 group. The improved efficacy in the combination group was associated with grade 3 or 4 adverse events in 59% of the patients.

There are a number of other checkpoint inhibitors, undergoing preclinical and clinical evaluation, that target receptors such as lymphocyte activation gene 3 protein (LAG-3), T cell immunoglobulin and mucin receptor-3 (TIM-3), V-domain Ig-containing suppressor of T cell activation (VISTA), and T cell immunoreceptor with Ig and ITIM domains (TIGIT) (9, 14).

### Other Agents That Modulate Immune Responses

Tumor-specific T cell responses may be increased by using agonists of co-stimulatory receptors on immune cells. Examples of receptors that could serve as targets for agonist antibodies are CD40, CD137, OX40, glucocorticoid-induced tumor necrosis factor receptor related protein (GITR) and inducible T cell co-stimulator (ICOS) (15). Anti-CD40 targets DCs and has been investigated in a few clinical trials; it has shown little activity as a single agent but may prove beneficial in combination therapies (16, 17). A problem with anti-CD40 is its toxicity but this may be overcome in the future (16, 17).

Toll-like receptor (TLR) agonists stimulate APCs, which in turn activate tumor-specific T cell responses. Some TLR agonists have failed in clinical trials because of the difficult balance between the anti-tumor immune responses and safety, for instance by indiscriminately activating the immune system. There are three approved TLR agonists in clinical settings: bacillus Calmette-Guérin (BCG), monophosphoryl lipid A (MPL) and imiquimod (18). These compounds are usually applied topically since they display disadvantageous toxic effects after systemic application.

Another approach is to target immunosuppressive mechanisms in the TME. Certain cytostatic drugs can eliminate or inactivate immune suppressor cells such as T_reg_ cells and myeloid derived suppressor cells (MDSCs). A typical example is low-dose cyclophosphamide that may preferentially target T_reg_ cells and allow for attenuation of T_reg_ (19).

## Local Tumor Destruction Methods

LTD methods such as radiofrequency ablation (RFA), microwave ablation (MWA), laser, cryotherapy, high intensity focused ultrasound (HIFU) and irreversible electroporation (IRE) have been used for decades as tools in the armamentarium against cancer, usually with palliative intent, but occasionally also with curative intent. Ionizing radiotherapy is usually not labelled as a local destruction method but is included here because of its widespread use.

### Basis for LTD-Induced Effects With Immunologic Consequences

There are several ways that local tumor destruction may induce an immune response that generates cytolytic effector cells: increase exposure and presentation of tumor antigens, make the TME more immunogenic (inflamed), including enhanced function APCs and lowered influence of tumor-associated immunosuppression, and reduce tumor burden (3, 20). It is likely that a strong immunologic anti-tumor effect requires the involvement of all or several of these factors (21–28).

LTDs have the potential to expose tumor antigens, including neoantigens, that have been hidden to the immune system (29, 30). They target the tumor directly without the need to predict and discover tumor-associated antigens.

LTDs may convert a non-inflamed (“cold”) tumor into an inflamed (“hot”) tumor, or increase the inflammatory component of an already inflamed tumor, based on the creation of danger-associated molecular pattern signals (DAMPs) and proinflammatory cytokines and antigens from dying tumor cells. This activates DCs, which facilitates antigen acquisition, processing and migration of the DCs to draining lymph nodes where they interact with potential effector cells. Examples of DAMPs are RNA, DNA, heat shock proteins (HSPs), high mobility group box 1 (HMGB1) protein, calreticulin, ATP and uric acid; typical pro-inflammatory cytokines are interleukin-1β (IL-1β), interferon-γ (IFN-γ), interleukin-6 (IL-6), interleukin-12 (IL-12), interleukin-8 (IL-8) and tumor necrosis factor-α (TNFα) (4, 20).

Necrotic cell death induces an inflammatory TME and is considered to be especially prone to trigger an immune response (4, 20, 31, 32). For instance, necrotic cells can produce mature DCs that are capable to induce antigen-specific T cells (31, 32). An immunogenic TME may also be created by an immunogenic form of apoptosis, called immunogenic cell death (ICD). ICD was first described for chemotherapeutics like anthracyclines, oxaliplatin and cyclophosphamide and has subsequently been shown to be induced also by radiotherapy and photodynamic therapy (PDT). It is characterized by endoplasmic reticulum stress and inflammatory changes and some of the key mediators appear to be, again, calreticulin, ATP, HSPs and HMBG1 (33, 34).

Tumors may escape immune attack by producing and releasing substances that decrease T cell function, like PD-L1 and PD-L-2. Other factors in the TME that favor immunosuppression are T_reg_ cells, MDSCs, hypoxia, tumor stroma cells (the so-called cancer-associated fibroblasts), nitric oxide, reactive oxidative species, vascular endothelial growth factor A (VEGF-A), indoleamine-2,3-dioxygenase (IDO), arginase, interleukin-10 (IL-10), prostaglandin E2 and transforming growth factor β (TGFβ) (4, 21, 22, 24). In the untreated tumor there is an imbalance between factors that promote and suppress an effective immune response against the tumor, usually favoring suppression. Likewise, while LTDs are intended to create an immunogenic TME this is often coupled with activation of suppressive mechanisms, and it is hoped that these opposing activities should tip the resulting balance in favor of anti-tumor immune activity.

Hyperthermic injury is the main mechanism for many of the methods in common use (RFA, laser, MWA, HIFU) and may be an important component in other methods such as IRE and photodynamic therapy (PDT). Most hyperthermic treatments use temperatures above 60°C, which causes protein denaturation and coagulation. This leads rapidly to coagulative necrosis, which destroys tumor antigens and endothelial cells. At temperatures lower than 60°C (and above 42°C), irreversible cell death, without instantaneous coagulation, occurs mainly due to inactivation of vital enzymes, and the time needed for cell death is longer the lower the treatment temperature (35–37). It has been shown *in vitro* that a temperature of 45°C leads to inactivation of vital enzymes and intracellular processes, which will lead to irreversible cell damage within 30 min (35). The tight relationship between temperature level and type of cell death was shown in another *in vitro* tumor system; heating for 30 min at 43, 43.5 and 44°C produced apoptosis, heating at 45°C produced a mixture of apoptosis and necrosis whereas heating at 46-47°C produced only necrosis (36). Studies *in vivo* using immunomodulating interstitial laser thermotherapy (imILT) showed that treatment at 46°C for 30 minutes ensured radicality in tumor-bearing rats (37).

Treatment at low temperatures is associated with a number of tissue events that favor antigen presentation and an immunologic response. The effects have been summarized by Hurwitz and include increased permeability of tumor vessels, increased expression of cancer antigens, increased expression of major histocompatibility complex (MHC) class I and II antigens, facilitated migration of APCs to lymph nodes, up-regulation of co-stimulatory molecules (e.g., CD80, CD86, CD40) on APCs with subsequent activation of T cells, up-regulation of the expression of toll-like receptor 4 on APCs, increased expression of intercellular adhesion molecule-1 (ICAM-1) which facilitates trafficking of T cells to peripheral tissue, and increased synthesis and surface expression of heat shock proteins (HSPs) in tumor cells (38).

### Evaluation Rationale

In this review, the focus is on studies that have investigated a possible abscopal effect of LTDs, without or with combination with immunotherapy. The associated immunologic changes in treated and untreated tissues in these studies have also been analyzed.

The “abscopal effect” is a term that was originally used to describe radiotherapy-induced tumor regression in lesions distant from a targeted site. Today, the term is used for regression of established distant untreated tumors after any form of local treatment. It is considered to be mainly immune mediated.

The term “lowered metastatic spread” is used here to denote decreased/absent spread of tumor in models where the effect on disease progression can be observed in fully comparable controls, i.e., in models where the primary, treated tumor has been eradicated, or reduced to the same extent, in the LTD-treated group and in the control group in order to eliminate the possible influence of continued metastatic spread from the treated tumor. With this definition, lowered metastatic spread is a strong indicator of a relevant immunologic effect.

“Rejection immunity” refers to lowered growth of a new challenging tumor and is considered to be a sign of immunologic memory. Rejection immunity is demonstrated in animals that are rechallenged some time after eradication of the first tumor, whereas the abscopal effect is demonstrated in animals that have a concomitant tumor that is untreated. Filatenkov et al. showed resistance against a second tumor when the first tumor had been eradicated a month earlier, whereas there was progressive growth of the second tumor when the challenge was performed at the time of treatment (39). Besides the difference in timing, one explanation is the difference in tumor burden at the time of challenge (40). The abscopal effect is studied under conditions that are closer to the human situation and its outcome should therefore be considered to be the strongest indicator of a relevant immunologic effect. Abscopal effect, lowered metastatic spread and rejection immunity are referred to as systemic effects.

### Literature Search

The electronic data bases PubMed and Embase were searched for abscopal effects after LTD treatment of tumors. Searches were restricted to the English language. The following query was used in PubMed:

(neoplasm OR cancer OR tumor) AND (radiofrequency ablation OR laser therapy OR photodynamic therapy OR microwave ablation OR thermotherapy OR high-intensity focused ultrasound OR cryotherapy OR electroporation OR electrochemistry OR radiotherapy) AND abscopal.

The terms used in Embase had small differences in wording with the intention to cover the same fields:

(neoplasm OR cancer OR tumor) AND (cryotherapy OR radiofrequency ablation OR laser surgery OR photothermal therapy OR laser induced thermotherapy OR laser induced thermal therapy OR photodynamic therapy OR microwave thermotherapy OR thermotherapy OR high intensity focused ultrasound OR electroporation OR electrochemotherapy OR radiotherapy) AND abscopal effect.

The reference lists of identified publications, including reviews, were checked for further references.

### Radiofrequency Ablation

In RFA a high-frequency alternating electric current is used. Heating is by ionic agitation within 2 mm of the probe surface and tissue heating beyond this is by heat conduction. A typical treatment produces temperatures of 60-100°C or more (41).

#### Systemic Effects

Systemic effects of RFA in various experimental models are summarized in **Table 1**. RFA alone produced an abscopal effect in 2/9 studies (44, 45, 47–53). Combination therapy with an immunomodulating regimen improved the effect in these two studies and resulted in an abscopal effect that was absent with RFA alone in six studies (44, 47, 49, 50, 52, 53). Rejection immunity could be demonstrated in seven studies following RFA alone (29, 42–44, 46, 51, 54).

**Table 1:** Effect on systemic disease after radiofrequency ablation (RFA) in animals.

Species/strain	Tumor model	RFA	Abscopal effect^a^	Rejection immunity^b^	Authors
**Mouse**					
C57BL/6n	B16-OVA	Alone		Yes (weak)	Den Brok et al, 2004, 2006 (29, 42)
		+ anti-CTLA-4 i.v. or i.p.		Yes	
C57BL/6	MB49	Alone		Yes	Dromi et al, 2009 (43)
		+ DC i.t.		Yes	
BALB/c	CT26-KS	Alone	No	Yes	Johnson et al, 2009 (44)
		+ huKS-IL2 i.t.	Yes	Stronger	
BALB/c	BNL IME A.7R.1	Alone	Yes		Iida et al, 2010 (45)
		+ EC1301 i.v.	Stronger		
C3H/HeJ	SCC7	Alone		Yes	Saito et al, 2011 (46)
		+ IL-2 gene transfer i.t.		Stronger	
CEA-Tg C57BL/6	MC38-CEA^+/-^	Alone	No		Gameiro et al, 2013 (47)
		+ poxviral vaccine s.c.	Yes		
C57BL/6	MC38	Alone	Yes		Nakagawa et al, 2014 (48)
		+ OK-432 *stim* DC i.t.	Stronger		
BALB/c	CT26	Alone	No		Shi et al, 2016 (49)
		+ anti-PD-1 i.p.	Yes		
BALB/c	CT26	Alone	No		Lemdani et al, 2019 (50)
		+ GM-CSF-BCG gel i.t.	Yes	Yes	
		+ GM-CSF-BCG gel i.t. + anti-PD-1 i.p.	Stronger		
**Rat**					
Wag/Rij	CC531	Alone	No	Yes	van Duijnhoven et al, 2005 (51)
**Rabbit**					
JWR	VX2	Alone	No		Hamamoto et al, 2013 (52)
		+ OK-432 i.t.	Yes	Yes	
JWR	VX2	Alone	No		Hamamoto et al, 2015 (53)
		+ BCG i.t.	Yes	Yes	
NZW	VX2	Alone		Yes	Behm 2016 (54)
		+ CpG s.c.		Stronger	

a Lowered growth of established distant tumor.

b Lowered growth of challenging tumor; i.v., intravenous; i.p., intraperitoneal; i.t., intratumoral; s.c., subcutaneous.

An abscopal effect was elicited or improved by various types of DC stimulating agents in five instances, using intratumoral injections of OK-432 (52), OK-432 stimulated DCs (48), BCG (50, 53) and BCG together with GM-CSF (50). Other agents that promoted an abscopal effect were huKS-IL-2 (tumor-specific monoclonal antibody fused to IL-2) (44), EC1301 (a chemokine agonist) (45) and anti-PD-1 (49).

An abscopal effect following RFA alone has been reported in four patients with renal cell carcinoma (55–57).

#### Immunologic Changes

RFA increased CD8 infiltration in treated tumors (45) and often in untreated tumors (45, 48–50). CD8 response, usually estimated by IFN-ƴ production, to tumor-specific stimulation was increased in regional lymph nodes (45, 46), sometimes in the spleen (44, 45, 48–50) but not in untreated tumor (48). Combination with immunotherapy either elicited, or increased, these CD8 parameters, regardless of the type of combination. The effect of RFA on T_reg_ cells and MSCDs has been studied in spleen and untreated tumor and changes have been minimal or absent (48–50), whereas combination with anti-PD-1 was associated with a decrease in T_reg_ cells in untreated tumor in one study (49).

##### Immunologic Changes in Patients

One study compared RFA in combination with intratumoral injection of autologous immature DCs with intratumoral DC injection alone in patients with advanced melanoma. The combination increased infiltration of CD8 and HSP expression and lowered infiltration of T_reg_ (CD4CD25) cells in treated tumor (58). The combined treatment increased time to progression but had no effect on overall survival.

Lemdani et al. treated liver metastases from colorectal carcinoma and found that RFA did not affect tissue levels of CD8 and T_reg_ lymphocytes in untreated liver tumors (50). In another study treating colorectal liver metastases, RFA was found to induce an increase of CD8 and PD-L1 in untreated primary colorectal tumor (49).

### Laser Thermotherapies

Lasers for photothermal therapy use wavelengths in the near infrared region since the absorption of light in biological tissue is relatively low at these wavelengths (59). The penetration of laser light is, however, only a few mm and the tissue is heated mainly by heat conduction (60).

Laser-induced thermotherapy (LITT) is usually used at constant, high output powers resulting in high temperatures and photocoagulation. Immune modulating interstitial laser thermotherapy, imILT, (previously called ILT) uses low power and a master temperature probe for feedback control of laser power to ensure a stable temperature and treatment precision (37, 61–63). Typically, the procedure aims at obtaining a temperature of 46°C at a chosen distance from the laser tip for a duration of 30 min. Laser immunotherapy (LIT) combines laser irradiation with local injection of glycated chitosan for activation of APCs, and laser irradiation is performed in a non-invasive mode for 10 minutes (64, 65).

Nanomaterials are increasingly being studied for selective absorption of light in order to enhance photothermal and photodynamic effects, often together with measures that strengthen the selectivity for cancer cells and/or local immune activity. They can also be designed as drug-delivery vehicles, e.g., in photodynamic therapy (PDT) (66, 67).

#### Systemic Effects

In preclinical studies, imILT and LITT, using a low laser output power (2 W), produced abscopal effects in 2/2 studies (68, 69) and lowered metastatic spread in 3/3 studies as compared to surgical resection when both methods eradicated the primary tumor (**Table 2**) (69, 78, 80). The results were obtained in two different rat liver adenocarcinoma metastases models. Rejection immunity was demonstrated in 5/5 studies (64, 70, 76, 77, 79).

**Table 2:** Effect on systemic disease after laser-induced thermotherapy in animals.

Species/strain	Tumor model	Laser method	Combined with	Abscopal effect^a^	Lowered metastatic spread	Rejection immunity^b^	Authors
**Mouse**							
C57BL/6	B16-F10	Low power				Yes	Dees et al, 2002 (70)
Balb/c	4T1	Nano-mediated		No	No		Wang et al, 2014 (71)
			+ anti-CTLA-4 i.v.	Yes	Yes		
C57BL/6	MB49	Nano-mediated		No			Liu et al, 2017 (72)
			+ anti-PD-L1 i.p.	Yes		Yes	
Balb/c	4T1	Nano-mediated	+ TLR7 ag incorp	No			Ge et al, 2018 (73)
			+ anti-PD-L1 i.v.	Yes			
Balb/c	4T1	Nano-mediated		Yes			Guo et al, 2019 (74)
			+ CpG ODN incorporation	Stronger			
Balb/c	4T1	Nano-mediated		No			Fu et al, 2020 (75)
			+ anti-PD-1 i.v.	Yes			
**Rat**							
Wistar F	DMBA-4	LIT				Yes	Chen et al, 1999, 2003 (64, 76)
Wistar F	DMBA-4	LIT, then ACT				Yes	Chen et al, 2001 (77)
Wistar F	DMH-CC	imILT			Yes		Möller et al, 1998 (78)
Brown Norwegian	BN7005	imILT		Yes			Tranberg et al, 2002 (68)
Wistar F	DMH-CC	imILT				Yes	Ivarsson et al, 2005 (79)
WAG	CC 531	LITT			Yes		Isbert et al, 2002 (80)
WAG	CC 531	LITT		Yes	Yes		Isbert et al, 2004 (69)

a Lowered growth of established distant tumor.

b Lowered growth of challenging tumor. LIT, laser interstitial thermotherapy; ACT, adoptive cell transfer; imILT, immunomodulating interstitial laser thermotherapy; LITT, laser-induced thermotherapy; i.v., intravenous; i.p., intraperitoneal.

Nanoparticle-based photothermal therapies have been shown to produce abscopal effects in 1/5 studies when used alone (71–75). An abscopal effect was regularly seen when photothermal therapy was combined with a checkpoint blocker (71–73, 75) and in a study where CpG ODN was incorporated into the nanomaterial (74).

imILT alone was shown to give a pronounced abscopal effect in one patient with advanced melanoma (81). LIT, combined with topical imiquimod (a TRL7 agonist), was reported to produce complete responses at non-treatment sites within the regional lymphatic drainage area in 4/11 patients with advanced melanoma (82). In another study, LIT was reported to give an abscopal effect in 1/10 patients with advanced breast carcinoma (83).

#### Immunologic Changes

Isbert et al. showed that LITT increased CD8 lymphocytes and B7-2 (CD68) expression at the invasion front of untreated twin tumors as compared to hepatic resection (69). In rechallenge experiments, the strong rejection immunity after imILT was associated with an immune cellular response of tumor-infiltrating macrophages and CD8 lymphocytes (79). Following laser thermotherapy, there are increased tissue levels of IFN- γ, IL-2 and IL-10 in treated tumors (70). Following nanoparticle-based photothermal therapy without any adjuvant, CD8 levels were increased in untreated tumors in 2/4 studies (71, 73–75), and T_reg_ cells increased in untreated tumor in one of two studies (71, 75). Combination with checkpoint blockade increased CD8 and lowered T_reg_ cells in these nanoparticle-based studies.

##### Immunologic Changes in Patients

The imILT-induced abscopal effect in a patient with advanced melanoma was associated with extensive necrosis and heavy infiltration of macrophages, CD3, CD4 and CD8 lymphocytes, CD11c and CD83 DCs and CD80 and CD86 in a large, untreated intraabdominal tumor. All these cell markers had been virtually absent in several biopsies obtained before imILT (81).

In patients with breast cancer, imILT induced an increase of CD8 lymphocytes within the tumor, an increase of mature CD83 DCs and CD20 B cells at the tumor border and a decrease of T_reg_ lymphocytes in regional lymph nodes (84). Vogl and coworkers reported that laser-mediated thermotherapy of colorectal liver metastases induced a tumor-specific T cell stimulation (CD4, CD8) and increased cytolytic activity of T cells against an allogenic tumor (85).

### Photodynamic Therapy

In photodynamic therapy (PDT) laser light excites a photosensitizer that generates reactive oxygen species, which kills tumor cells by various mechanisms, leading to cell death by necrosis and/or apoptosis (86).

#### Systemic Effects

Following PDT alone an abscopal effect was demonstrated in 3/10 animal studies (87–96), including six recently performed studies that used nanoparticles loaded with a photosensitizer (91–96) and occasionally also with a chemotherapeutic agent (93, 95). Lowered metastatic spread was seen in one study that used a two-step treatment regimen, based on first administering a low light dose and then a high light dose (97). Rejection immunity after PDT alone was observed in three studies (87, 89, 97).

Combination with intratumoral injection of DCs produced an abscopal effect in two studies (88, 98). Nanoparticle-mediated PDT always produced abscopal effects when combined with checkpoint blockade (92–96). The Hamblin group found that low dose cyclophosphamide before PDT induced rejection immunity in two different experimental models (99, 100).

There is one report of an abscopal effect following repeated treatments with PDT in a patient with recurrent angiosarcoma (101).

#### Immunologic Changes

Following PDT alone, increased tumor-specific cytotoxic T cell activity has been observed in treated tumor (89), regional lymph nodes (89, 97) and untreated tumor (89) but not in the spleen (88, 93). Increased cytotoxic T cell activity was, however, seen in the spleen when PDT was combined with intratumoral DCs or systemic cyclophosphamide or anti-PD-L1 (88, 93, 98, 99). The effect on CD8 levels have been studied mainly in untreated tumor, where they were unaffected by PDT alone and increased after combination with a checkpoint blocker (92–94).

Increased levels of T_reg_ cells in regional lymph nodes, spleen and untreated tumor have been reported following PDT alone. These changes in T_reg_ levels normalized or reversed following combination with low dose cyclophosphamide or checkpoint blockade (94, 100).

##### Immunologic Changes in Patients

In patients treated with PDT for basal cell carcinoma (BCC), enhanced recognition and reactivity of blood lymphocytes against Hip1, a known BCC-associated tumor antigen, was demonstrated, which indicated increased systemic immune response against the tumor (102).

### Microwave Ablation

In MWA, a high-speed electromagnetic field forces rotation of water molecules. MWA can heat tissue up to 2 cm away from the antenna and temperatures are typically 60-100°C or more (103).

#### Systemic Effects

In two different models of murine carcinoma an abscopal effect was absent when MWA was given alone but was seen when it was combined with intratumoral administration of GM-CSF and IL-2 (104) or with intratumoral GM-CSF together with intraperitoneal anti-CTLA-4 (105). Rejection immunity after these treatments displayed a similar pattern.

#### Immunologic Changes

In cured mice, the combination treatments were shown to induce tumor-specific T lymphocytes in the spleen. These changes were not seen after MWA alone (104, 105).

##### Immunologic Changes in Patients

In 82 patients with hepatocellular carcinoma, Dong et al. found that MWA increased tissue levels of CD3, CD56 and CD68 in the treated tumor. These markers increased also in an untreated tumor, located in a different liver lobe than the target tumor, although to a smaller extent and unaccompanied by an abscopal effect (106).

### Magnetic Thermotherapy

In magnetic hyperthermia, the tumor is heated by exposure to an alternating magnetic field following injection of metallic nanoparticles or insertion of thermoseeds into tumor. Magnetic hyperthermia gives the possibility to achieve similar temperatures throughout the whole tumor (107, 108).

#### Systemic Effects

Using magnetic nanoparticles or thermoseeds, an abscopal effect following magnetic thermotherapy alone has been demonstrated in 3/3 animal studies (107–109). In these studies, the goal was to attain temperatures at 55°C or lower throughout the whole treated tumor during 10-30 min. Rejection immunity was demonstrated in 2/2 of these studies.

#### Immunologic Changes

Toraya-Brown et al. demonstrated increased percentage of CD8 and increased expression of CD80 and CD86 on DCs in regional lymph nodes (107). In a rat study, CD4, CD8 and NK cells were demonstrated to increase in treated and untreated tumor, together with specific cytotoxic T cell activity in the spleen (109).

### High Intensity Focused Ultrasound

In HIFU an array of high energy ultrasound beams are targeted on a selected area, which gives a temperature rise and acoustic cavitation with mechanical disruption. HIFU is usually used in a thermal ablative mode that results in rapid coagulation necrosis, but the tissue effects can be changed by changing the sonication parameters to get predominantly heat generation or mechanical disruption (histotripsy) effects (110, 111).

#### Systemic Effects

HIFU has been shown to promote abscopal effects when combined with CpG + anti-PD-1 and anti-CTLA-4 + anti-PD-L1, but not when used alone (112–114). HIFU alone has been shown to induce rejection immunity in 2/2 studies (115, 116).

#### Immunologic Changes

After HIFU alone, there were increased levels of CD8 in the treated tumor, but not in untreated tumor (112, 114), together with increased levels of PD-L1 in treated tumor (114). Other findings of interest following HIFU alone were that DCs increased in regional lymph glands and spleen and that T_reg_ cells decreased in treated tumor and regional lymph glands but not in untreated tumor (112, 114). In another study, HIFU alone induced increased tumor-specific cytolytic activity in splenic lymphocytes (116).

##### Immunologic Changes in Patients

Wu and coworkers treated women with breast cancer with HIFU followed by modified radical mastectomy 1-2 weeks later and compared the results with mastectomy alone. They found that HIFU increased infiltration of activated APCs (DCs and macrophages) and CD3, CD4, CD8 and B lymphocytes and NK cells in the margins of the treated tumors (117, 118).

### Cryotherapy

The mechanism by which cryotherapy results in tumor cell death is complex and the effect depends on final tissue temperature, the rate of freezing, duration of freezing, the rate of thawing, and the number of freeze-thaw cycles (119, 120). Cryotherapy maintains the cell structure and kills cells without denaturating intracellular tumor antigens.

#### Systemic Effects

A summary of experimental studies where cryotherapy has been shown to have systemic effects is given in **Table 3**. An abscopal effect following cryotherapy alone was demonstrated in 1/4 studies (121, 124, 125, 129), whereas an abscopal effect was elicited in 4/4 studies when cryotherapy was combined with systemic anti-CTLA-4 or with different methods of dendritic cell stimulation such as CpG containing oligodexoynucleotide (CpG-ODN), lipopolysacharide (LPS), and DCs stimulated with Bacillus Calmette-Guerin cell wall skeleton (BCG-CWS) (124, 125, 129, 130). Lowering of metastatic spread was observed in one study using cryotherapy alone (121), in another study only when a high rate of freeze was used (120) and in one instance first after combination with intratumoral administration of DCs (123). Rejection immunity after cryotherapy alone was reported in 5/7 studies (42, 122–124, 126–128).

**Table 3:** Effect on systemic disease after cryotherapy in animals.

Species/Strain	Tumor	Cryotherapy	Abscopal effect^a^	Lowered metastatic spread^a^	Rejection immunity^b^	Authors
**Mouse**						
BALB/c	26-B	Alone	Yes			Joosten et al, 2001 (121)
BALB/c, nude	MV3	Alone		Yes		
BALB/c	MT-901	Alone			Yes	Sabel et al, 2005 (122)
BALB/c	4T1	Alone- High rate of freeze		Yes		Sabel et al, 2010 (120)
		- Low rate of freeze		No		
C57BL/6	3LL	Alone		No	No	Machlenkin et al, 2005 (123)
		+ DC i.t.		Yes		
	B16-OVA	Alone			No	
		+ DC i.t. + ACT i.v.			Yes	
C57BL/6n	B16-OVA	Alone			Yes	Den Brok et al, 2006 (42)
		+anti-CTLA-4/anti-CD25 i.p.			Stronger	
C57BL/6n	B16-OVA	Alone			Yes	Den Brok et al, 2006 (124)
		+ CpG-ODN p.t.			Stronger	
	B16F10	Alone	No			
		+ CpG-ODN p.t.	Yes			
BALB/c	CT26	Alone	No			Udagawa et al, 2006 (125)
		+ BCG-CWS stimul DCs i.t.	Yes			
C57BL/6	B16-OVA	Alone			Yes	Redondo et al, 2007 (126)
		+ topical Imiquimod			Stronger	
BALB/c	CT26	Alone			No	Levy et al, 2009 (127)
		+ cyclophosphamide i.p.			Yes	
C57BL/6n	B16-OVA	Alone			Yes	Nierkens et al, 2009 (128)
		+ CpG-ODN i.v.			Yes	
		+ CpG-ODN p.t.			Stronger	
C57BL/6	TRAMP C2	Alone	No			Waitz et al, 2011 (129)
		+ anti-CTLA-4 i.p.	Yes			
C57BL/6	LL2	+ LPS p.t.	Yes			Takahashi et al, 2016 (130)

a Lowered growth of established distant tumor.

b Lowered growth of challenging tumor. I.v., intravenous; i.p., intraperitoneal; p.t., peritumoral.

There is one report of a possible abscopal effect in a patient receiving cryotherapy together with intratumoral anti-PD-1 in renal cell cancer (131).

#### Immunologic Changes

The number and tumor-specific activities of CD8 cells after cryotherapy alone sometimes increased in regional lymph glands but stayed unchanged in spleen and untreated tumor, whereas combinations with different immunomodulators resulted in increases in these CD8 parameters (42, 120, 122, 123, 125–129). Cryotherapy has been shown to increase antigen loading and activation and maturation of DCs in regional lymph nodes (42, 124).

Sabel et al. reported that the levels of CD4CD25 cells in regional lymph nodes decreased when cryotherapy was given at a high rate of freeze and increased when cryotherapy was performed with a low rate of freeze, which correlated with the effect on metastatic spread (120). Waitz et al. found that T_reg_ levels stayed unchanged in untreated tumors after adding intraperitoneal anti-CTLA-4, which together with increased levels of CD8 led to an increased CD8/T_reg_ ratio (129). A similar finding of increased CD8 together with unchanged T_reg_ levels in untreated tumor following cryotherapy + LPS has been reported (130).

### Irreversible Electroporation

IRE generates short (microseconds to milliseconds) electric high-voltage pulses across cell membranes that cause increased cell permeability and cell death. The extracellular matrix, collagen structures, blood vessels and bile ducts remain intact. Heat is not considered to be the dominant cytotoxic mechanism (132), but it has been shown that increases in temperature may be substantial and cause tissue effects, especially when pulse length and probe exposure length are long (133, 134). Because the majority of the proteins in the electrical field are not denatured in IRE, the tumor antigens that are left in the ablated tissue should remain intact.

IRE alone was unable to produce an abscopal effect in a pancreatic cancer model. The outcome was the same when it was combined with either anti-PD-1 or a TLR7 agonist, whereas an abscopal effect could be demonstrated when it was combined with both these immunomodulators (135). Rejection immunity was demonstrated after IRE alone, confirming a previous experimental study (136). The positive combination study was associated with increased CD8 levels and unchanged T_reg_ levels in untreated tumor (135).

### Reversible Electroporation

Like IRE, reversible electroporation uses short electric high-voltage pulses across cell membranes. The difference is that the voltage and the frequency are chosen to transiently increase the permeability of cell membranes, which allows normally non-permeant molecules to enter the cytoplasm and the cell nucleus. When a cytostatic drug is used this form of electroporation is called electrochemotherapy (ECT) (137). Bleomycin and cisplatin are the most common chemotherapeutic agents in ECT. The technology can also be used to deliver plasmid DNA into a target tumor and is then called gene electrotransfer (GET) (138).

#### Systemic Effects

Reversible electroporation alone did not produce an abscopal effect (139–143). This was also the case for ECT using bleomycin (144, 145), whereas ECT with cisplatin produced an abscopal effect (143). The combination of ECT with peritumoral injections of allogenic cells secreting IL-2 or intratumoral injections of CPG ODN gave an abscopal effect (144, 145). Rejection immunity was observed in 3/4 studies following ECT alone (145–148); it was also seen in one study using calcium as the internalized agent (146).

With the GET approach, an abscopal effect was seen in 8/8 studies, using plasmids encoding IL-12 (141, 142, 149–151), GM-CSF/B7-1 (139, 152) and anti-CTLA-4 antibodies (140). Rejection immunity was demonstrated in 5/5 studies following GET (139, 140, 149, 151, 152). After adding intraperitoneal anti-CD25 to the GM-CSF/B7-1 GET regimen, it was also shown that no tumors developed in naïve mice that received i.v. injections of splenocytes from cured animals together with tumor cells (153).

Electroporation with plasmid IL-12 delivery has been reported to induce complete regression of distant, untreated cutaneous melanoma lesions in 2/19 (10%) patients (154). This response developed over a span of 6-18 months.

#### Immunologic Changes

ECT with bleomycin or cisplatin resulted in increased infiltration of CD8 lymphocytes and DCs in the treated tumor (143–145). When ECT was combined with CPG ODN, increased numbers of OVA-specific CD8 cells were demonstrated in regional lymph nodes together with an increased T cell IFN-*γ* response against ovalbumin in the spleen (145). When ECT was combined with peritumoral injections of allogenic IL-2 secreting cells, an increased infiltration of CD8 lymphocytes was found in the untreated tumor (144).

Electroporation with gene transfer has been shown to increase CD8 levels in treated tumor (149, 152, 153) and to produce tumor-specific CD8 cells with increased cytolytic activity and IFN-ƴ production in treated tumor, spleen and untreated tumor (139, 142, 151, 153). In patients, increased infiltration of CD8 lymphocytes has been observed in treated tumors after this kind of treatment (154).

### Radiotherapy

Radiotherapy produces DNA damage, which may lead to cell death by mechanisms such as senescence (permanent proliferative arrest), mitotic catastrophe, apoptosis, necroptosis and necrosis. The anti-tumor immune responses depend primarily on necrosis, necroptosis and apoptosis to produce forms of immunogenic cell death that are accompanied by an increase in MHC1 expression, release of DAMPs and an inflammatory environment (155).

#### Systemic Effects

A possible abscopal effect was investigated in 48 studies (**Table 4**) (39, 156, 157, 159–201). It could be demonstrated in 7/48 (15%) studies when radiotherapy was used alone (162, 164, 166, 168, 173, 183, 197). Radiotherapy in combination with an immunomodulating agent produced an abscopal effect in all but two of 45 studies; it failed in one study using radiotherapy and IL-2 against a weakly immunogenic tumor (SL2) (156), and in one study where combination with anti-PD-1 resulted in an abscopal effect in an immunogenic tumor (T1) but not in a non-immunogenic tumor (B16-F10) (191).

**Table 4:** Effect on systemic disease after radiotherapy in animals.

Species/Strain	Tumor	Combined with	Abscopal effect^a^	Rejection immunity^b^	Authors
**Mouse**					
DBA/2JIco	SL2	Alone x1	No		Everse et al, 1997 (156)
		+ rIL-2 p.t.	No		
BALB/c	67NR	Alone x1	No		Demaria, 2004 (157)
		+ Flt3-ligand i.p.	Yes		
C3Hf/KamLaw	Fibrosarcoma	Alone x1 or 10		Yes	Mason et al, 2005 (158)
		+ CpG-ODN p.t., i.t.		Stronger	
C3H/He	SCCVII	Alone x3	No		Akutsu et al, 2007 (159)
		+ DC i.t.	Yes		
BALB/c C57BL/6	Colon 26 LLC	Alone x1	No		Shiraishi et al, 2008 (160)
		+ ECI301 i.v.	Yes		
BALB/c C57BL/6	TSA MCA38	Alone x3, x5	No		Dewan, 2009 (161)
		+ anti-CTLA-4 i.p.	Yes		
BALB/c	Colon 26	Alone x5x2	Yes		Yasuda et al, 2011 (162)
		+ IL-2 i.t.	Yes (stronger)		
BALB/c	TSA	Alone x3	No		Dewan et al, 2012 (163)
		+ imiquimod topical	Yes	Yes	
		+ imiquimod topical + cyclophosphamide i.p.		Yes	
CEA-Tg C57BL/6	MC38-CEA^+/-^	Alone x1	Yes		Hodge, 2012 (164)
		+ poxvirus-based CEA vaccine	Yes (stronger)		
	LL2-CEA^+^	Alone	No		
		+ poxvirus-based CEA vaccine	Yes		
BALB/c C57BL/6	TUBO MC38	Alone x1	No		Deng et al, 2014 (165)
		+ anti-PD-L1 i.p.	Yes	Yes	
C57BL/6	LLC1	Alone x1	Yes		Kanagavelu et al, 2014 (166)
					
BALB/c C3H/HeN	Colon26 FM3A	Alone x1	No		Kanegasaki et al, 2014 (167)
		+ EC1301 i.v.	Yes		
C57BL/6	EL4	Alone x 1	Yes	Yes	Yoshimoto et al, 2014 (168)
BALB/c	CT26	Alone x 1	No		Young et al, 2014 (169)
		+ TGFβ inhibition p.o.	Yes	Yes	
BALB/c	CT26	Alone x1	No	Yes	Filatenkov et al, 2015 (39)
BALB/c C57BL/6	RENCA B16-OVA	Alone x1	No		Park et al, 2015 (170)
		Anti-PD-1 ± anti-CTLA-4 i.p.	Yes		
C57BL/6 BALB/c	B16-F10 TSA	Alone x1	No		Twyman-Saint Victor et al, 2015 (171)
		+ anti-CTLA-4 i.p.	Yes		
		+ anti-CTLA-4 + anti-PD-1 i.p.	Yes (stronger)	Yes	
BALB/c	4T1, TSA	Alone x5	No		Vanpouille-Box et al, 2015 (172)
		+ anti-TGFβ i.p.	Yes		
		+ anti-TGFβ + anti-PD-1 i.p.	(Yes for survival)		
BALB/c	Mesothelioma AB12	Alone x3	Yes		Wu et al, 2015 (173)
		+ anti-CTLA-4 i.p.	Yes (stronger)		
C57BL/6 BALB/c	MC38, B16-OVA 4T1	Alone x3	No		Rodriguez-Ruiz et al, 2016 (174)
		+ anti-CD137 i.p.	No (MC38), Yes (B16-OVA, 4T1)		
		+ anti-PD-1 i.p.	No (MC38), Yes (B16-OVA, 4T1)		
		+ anti-CD137+ anti-PD-1 i.p.	Yes (all)	Yes	
BALB/c	CT26	Alone x1		Yes	Young et al, 2016 (175)
		+ anti-CTLA-4 i.p.		Yes	
		+ anti-OX40 i.p.		Yes	
BALB/c C57BL/6	CT26 4434	Alone x5	Unusual		Dovedi et al, 2017 (176)
		+ anti-PD-1 i.p.	Yes	Yes	
		+ anti-PD-L1 i.p.		Yes	
NSG	A204	Alone x1	No		Eckert et al, 2017 (177)
humanized		+ NHS-IL12 i.v.	Yes		
C57BL/6	MOC1	Alone x2, x10	No		Morisada et al, 2017 (178)
		+ anti-PD-1 i.p.	Yes		
C57BL/6	MC38	Alone x3	No		Rodriguez-Ruiz et al, 2017 (179)
		+ anti-CD137 i.p.	No		
		+ anti-PD-1 i.p.	No		
		+ anti-CD137+ anti-PD-1 i.p.	Yes		
BALB/c	4T1	Alone x1	No		Schrand et al, 2017 (180)
		+ anti-CTLA-4 i.p.	Yes		
		+ anti-PD-1 i.p.	No		
		+ anti-CTLA-4 + anti-PD-1 i.p.	No		
		+ VEGF-4-1BB aptamer conjug i.v.	Yes		
C3H/HeNJcl	LM8	Alone x1	No		Takahashi et al, 2017 (181)
		+ IL-2/S4B6 i.p.	Yes		
C57BL/6	TC-1	Alone x 8	No		Chang et al, 2018 (182)
		+ DNA vaccine	Yes		
C57BL/6	LL/2, B16F10	Alone x 9	No		Lan et al, 2018 (183)
		Alone x 2	Yes		
BALB/c	CT26, TUBO	Alone x 6 with nMOF i.t.	No		Lu et al, 2018 (184)
		+ IDO inhibitor i.t., i.v.	Yes	Yes	
C57BL/6NTac	LLC1	Alone x 1	No		Moreau et al, 2018 (185)
		+ anti-CD40 i.t.	Yes		
129Sv/Ev	344SQ	Alone x3	No		Niknam et al, 2018 (186)
		+ anti-OX40 i.t.	Yes		
C57BL/6J	KPC	Alone x 1	No		Rech et al, 2018 (187)
		+ anti-PD-1/anti-CTLA-4 i.p.	No		
		+ anti-PD-1/anti-CTLA-4 + anti-CD40	Yes		
BALB/c	C51	Alone x 1 or 2 or 5	No		Rekers et al, 2018 (188)
		+ L19-IL-2 i.p.	Yes		
129Sv/Ev	344SQ	Alone x 3	No		Schoenhals et al, 2018 (189)
		+ anti-PD1 i.p.	Weak		
		+ anti-PD1+ anti-GITR i.p.	Yes		
C57BL/6	Panc02, KPC	Alone x 1	No		Yasmin-Karim et al, 2018 (190)
		+ anti-CD40 i.t.	Yes		
C57BL/6 BALB/c	B16-CD133 4T1	Alone x 3 or 5	No		Zhang, Niedermann, 2018 (191)
		+ anti-PD-1 i.p.	Yes		
C57BL/6	B16-F10	Alone x 3 or 5	No		
		+ anti-PD-1 i.p.	No		
129Sv/Ev	344SQ	Alone x 3	No		Caetano et al, 2019 (192)
		+ anti-PD-1 i.p.	No		
		+ anti-PD-1 i.p. + anti-MerTK i.p.	Yes		
C57BL/6JRj	AT-3	Alone x1	No		Kroon et al, 2019 (193)
		+ anti-PD-1 i.p + anti-CD137 i.t.	No		
		+ anti-PD-1 i.p. + anti-CD137 i.t. + cisplatin i.v.	Yes		
C57BL/6	B16-CD133, MC38	Alone x 2	No		Luo et al, 2019 (194)
		+ anti-PD-1 i.p.	Yes		
		+ anti-PD-1 i.p. + cisplatin i.p.	Yes (stronger)		
C57BL/6	B16-F10, DM4	Alone x 4	No		Pfannenstiehl et al, 2019 (195)
		+ anti-PD-1 i.p.	Yes		
C57BL/6	LLC	Alone x 3	No		Wang et al, 2019 (196)
		+ anti-PD-L1 i.p.	Yes		
C57BL/6	MC38, B16-F10	Alone x3	Yes (moderate)		Baba et al, 2020 (197)
		+ anti-PD-1 i.p.	Yes (stronger)		
C57BL/6	CT26	Alone x 2	No		Chen B et al, 2020 (198)
		+ IDO-inhibitor p.o.	Yes		
129Sv/Ev	344SQ	Alone x 3	No		Chen D et al, 2020 (199)
		+ anti-PD-L1 i.p.	No		
		+ anti-PD-L1 i.p. + anti-SHP-2 p.o.	Yes		
C57BL/6	LLC	Alone x 1	No	No	Liang et al, 2020 (200)
		+ VEGFR2 inhibitor p.o.	Yes	Yes	
C57BL/6	TC-1	Alone x 1	No	No	Wood et al, 2020 (201)
		+ anti-CD40 i.t.	Yes	Yes	

a Lowered growth of established distant tumor.

b Lowered growth of challenging tumor. i.v., intravenous; i.p., intraperitoneal; i.t., intratumoral; p.t., peritumoral; s.c., subcutaneous; p.o., peroral.

The combination with one or both of the checkpoint blockers anti-CTLA-4 and anti-PD-(L)1 was demonstrated to give an abscopal effect in 21/22 studies; however, in 6 cases it was observed first when anti-CD137 (179), a cytostatic drug (cisplatin) together with anti-CD137 (193), anti-CD40 (187), anti-GITR (189) or a tyrosine kinase inhibitor (192, 199) was added to the protocol. It is noteworthy that the latter three studies were performed on tumors based on an anti-PD-1 resistant cell line (189, 192, 198). In one study combination with anti-CTLA-4 produced an abscopal response whereas anti-PD1, alone or in combination with anti-CTLA-4, did not (180).

Rejection immunity was investigated in relatively few studies and was seen in 4/6 studies after radiotherapy alone (39, 158, 168, 175, 200, 201) and in 11/11 studies after combined treatment.

Despite the extensive use of radiotherapy in cancer therapy, an abscopal effect after radiotherapy alone has rarely been observed in patients, witnessed by the 41 cases found in a 2016 review that covered more than four decades (202). An interesting observation is that partial irradiation (using 1-3 fractions) of hypoxic regions of bulky lung tumors led to improved local control and abscopal effects as compared to conventional, whole-tumor fractionated radiotherapy (203).

The experience from combining radiotherapy with immunotherapy is steadily increasing, and a number of retrospectively analyzed case series suggest that radiotherapy can induce an abscopal effect when it is combined with anti-CTLA-4, anti-PD-1 or GM-CSF. Most studies have been performed in patients with melanoma. Radiotherapy was given after a period of treatment with anti-CTLA-4 or anti-PD-1 in six studies (204–209). In these studies, it was reported that the presumed radiotherapy-induced abscopal response rate was 52% (205), 25% (204), 27% (206), 19% (207) and 29% (209) or that the overall response rate was 24% (208). Radiotherapy was started before and/or concurrent with immunotherapy in five studies, using anti-CTLA-4 (n=3), anti-PD-1 (n=1) or CM-CSF (n=1) (171, 210–213), and in these studies the systemic effects were reported as abscopal response rates of 27% (210) and 18% (171), as complete response rates of 5% (210), 25% (211) and 2% (213) and as overall response rates of 45% (212) and 9% (213). The outcome was not obviously different for the two timing protocols or the different immunotherapeutic agents, whereas it appeared to be better for melanoma patients than for patients with other malignant diseases.

#### Immunologic Changes

Following radiotherapy alone, changes in CD8 levels in the treated tumor have been inconsistent, i.e., CD8 cells have been reported to increase (164, 180, 190, 196, 198, 199), stay unchanged (172, 173, 182, 186, 191, 195) or decrease (174, 176). In contrast, CD8 levels rose in combination therapies regardless of the immunomodulating agent used. In the spleen, CD8 levels, as estimated by tumor antigen specific activity, were unchanged following radiotherapy alone and usually increased in combination with an immunostimulant (157, 161, 182, 191, 200). Radiotherapy alone did not usually affect tissue levels of CD8 in untreated tumors (160, 161, 172, 176, 195, 196, 198–200, 214, 215) but both an increase and a decrease have been reported (173, 174, 190, 197). Combination with immunotherapy, regardless of the type, always increased CD8 levels in untreated tumors in these studies.

T_reg_ cells increased or stayed unchanged in the treated tumors following radiotherapy alone, and the levels were only marginally different after combination therapies (165, 173, 174, 180, 182, 198, 199). In the untreated tumor, T_reg_ levels were usually unaffected by radiotherapy alone and remained so, or decreased slightly, after combination with immunotherapy (173, 174, 181, 195, 198, 199). MDSC levels did not show a consistent pattern after radiotherapy and changed little, or not at all, after adding an immunomodulating agent in treated and untreated tumors (165, 174, 176, 190, 196).

The levels of PD-L1 in tumor and immune cells were increased in the treated tumor and sometimes also in the untreated tumor cells after radiotherapy alone (165, 170, 172, 174, 176, 196, 200). After combination with an immunomodulating agent, usually anti-PD-(L)1, the changes in PD-L1 levels have been variable both in irradiated and non-irradiated tumors (172, 176, 196, 200). PD-1 levels in CD8 cells have either stayed unchanged or increased in treated and untreated tumors after radiotherapy (165, 170, 172, 174, 196).

## Discussion

### Effects of Local Tumor Destruction

LTDs in combination with immunotherapy regularly produced abscopal effects in the preclinical studies. It was less common after LTD alone with one notable exception: methods that produced local hyperthermia at relatively low tissue temperatures, around 55°C at the tumor border, or less, regularly produced an abscopal effect and/or lowered metastatic spread (**Table 5**). This was accomplished by temperature feedback control with the imILT method (68, 78) and by careful selection of laser power and time with the LITT method (69, 80). It was also accomplished using magnetic hyperthermia where the goal was to attain temperatures at 55°C or lower throughout the whole tumor (107–109).

**Table 5:** Portion of systemic effects after LTDs in preclinical studies.

Method	Abscopal effect, lowered metastatic spread	Rejection immunity
		
RFA alone	2/9	7/7
+ immunomodulation	8/8	8/8
		
Laser at low temperatures	4/4	5/5
		
Nano-particle based photothermal therapy	1/5	–
+ immunomodulation	5/5	1/1
		
Photodynamic therapy (PDT) alone	3/11	4/4
+ immunomodulation	7/7	3/3
		
Microwave ablation (MWA) alone	0/2	0/2
+ immunomodulation	2/2	2/2
		
Magnetic thermotherapy at low temperatures	3/3	2/2
		
High intensity focused ultrasound (HIFU) alone	0/1	2/2
+ immunomodulation	1/1	3/3
		
Cryotherapy alone	3/7	5/7
+ immunomodulation	4/4	6/6
		
Irreversible electroporation (IRE) alone	0/1	1/1
+ immunomodulation	1/1	1/1
		
Reversible electroporation alone, incl ECT alone	1/7	3/4
+ immunomodulation, incl gene electrotransfer	10/10	7/7
		
Irradiation alone	7/48	4/6
+ immunomodulation	43/45	11/11
		

For references, see text and **Tables 1**–**4**.

These studies with low temperature level thermotherapy have been performed in mice and rats, and the tumors were inoculated/implanted subcutaneously or into the liver and the read-out (untreated) tumors were situated at one of these two sites. Abscopal effects were obtained for chemically induced (68, 69, 78, 80, 107, 109) and spontaneous (107, 108) tumors and in tumors that are considered to be immunogenic (107, 109) and poorly immunogenic (68, 69, 78, 80, 107).

**Figure 1:**
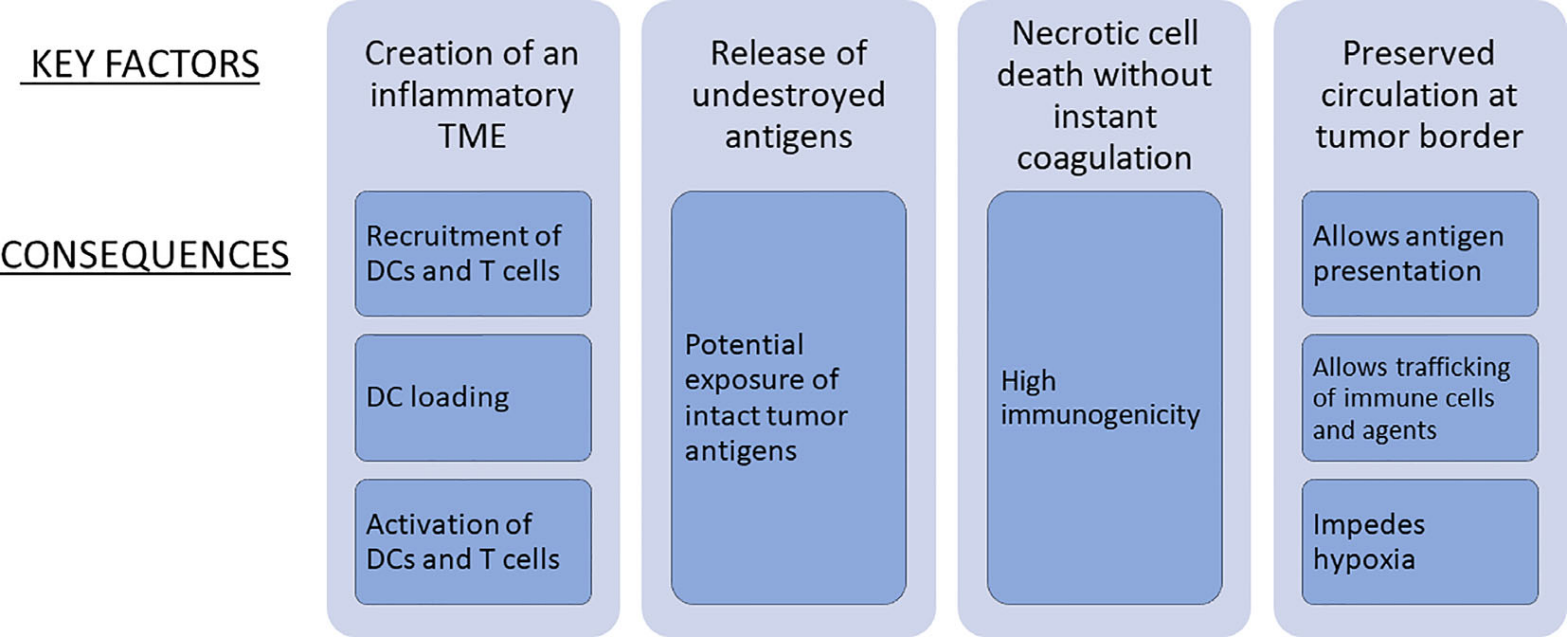
Key factors for an abscopal effect of LTD when used alone and in combination with immunotherapy.

So, the question arises, why did the LTDs differ in their ability to produce abscopal effects and why, in particular, could low level hyperthermia consistently produce such an effect on its own? The following analysis examines some of the key factors for an abscopal effect following LTD, such as the immune characteristics of the TME, and other factors that are important for the initiation, quality and strength of the immune response such as antigen exposure, type of tumor cell death and blood flow and tissue perfusion, factors that are closely interrelated (**Figure 1**).

#### Tumor Microenvironment

An immunogenic milieu triggers the uptake of tumor antigens by APCs and is characterized by infiltration of TILs, especially CD8 lymphocytes, a high ratio of CD8 over Foxp3 regulatory T cells, PD-L1 expression, a relatively high number of somatic mutations, MHC1 expression, and tertiary lymphoid structures. A non-inflamed tumor lacks some or several of these traits (21, 24, 25, 216). Immunologic changes in tissue that have correlated best with clinical and preclinical success after various immunotherapies are increased tissue levels of CD8 lymphocytes and mature DCs, lowered levels of Foxp3 cells and an increased CD8/Foxp3 ratio (3, 21, 25). It has been shown that checkpoint inhibitors and vaccine therapies work best when the tumor is T cell inflamed (24, 25, 217). A major action of LTDs is to increase the inflammatory component of the tumor.

In the reviewed studies, treatments without combination with an immunomodulating agent were usually followed by increased levels of CD8 in the treated tumor, with the exception of radiotherapy where the outcome was quite variable with even a few instances of a decrease in CD8. An increase of CD8 in untreated tumor was often seen after RFA, low level hyperthermia and reversible electroporation, whereas it was a rare event following radiotherapy. An increase in DCs was usually seen in treated tumor and/or regional lymph glands. T_reg_ cells stayed unchanged or increased at most sites regardless of treatment type. Changes of MDSCs, PD-1 and PD-L1 have been studied mainly in the radiotherapy studies. The changes in MDSCs and PD-1 have not shown any uniform pattern, whereas PD-L1 increased consistently in treated tumor and sometimes also in untreated tumor.

While all LTDs generated inflammatory changes, it is obvious that the reported changes in immunologic parameters do not suffice to explain why there was such a large variation in the ability to produce abscopal effects. The use of many different tumor models and the heterogeneity and complexity of models and design makes it difficult to compare the efficacy of different LTDs. Another difficulty is that the changes in the TME are time-dependent, which infers that the time-points chosen for analysis might have missed or been insufficient for finding changes that are relevant for anti-tumor activity. For instance, in a study using high single dose irradiation, it was observed that CD8 cells showed a transient decrease followed by an increase, whereas MDSCs showed an increase followed by a decrease in the irradiated tumor (39).

#### Antigen Exposure

The likelihood for an anti-tumor immune response increases with mutational load, which indicates that increased tumor antigen exposure would increase anti-tumor immunity and response to immunotherapy (218). Melanoma, NSCLC and tumors with mismatch repair deficiency contain a relatively large number of mutations, and patients with these tumors are more likely to respond to checkpoint inhibitors than others (23, 26, 219). There is, however, generally no correlation between the density of tumor antigens and the degree of T cell infiltration in the TME (27). The importance of a concomitant inflammatory milieu with antigen-specific CD8 cells for immune efficacy has been shown in untreated patients and patients treated with anti-PD-1 (28, 217).

It should also be remembered that only a minute part of mutations is recognized by T cells in the tumor-bearing patient (218). In advanced melanoma, Rosenberg and Restifo found that only a small fraction of nonsynonymous mutations led to a neoantigen that was detected by tumor-infiltrating lymphocytes (TILs). Furthermore, they found that every mutation recognized by TILs was unique and not shared by other melanomas in the studied population of 21 patients (7).

The ability of LTDs to release antigens may increase the number and diversity of responding T cells (29, 30). The resulting activation signal may, however, be weak or absent if the antigens released by the LTD are coagulated or otherwise destroyed. When treatment is performed at low temperature levels, for instance with the feedback imILT protocol, there is a zone with temperatures that are below coagulation threshold and contains cells that maintain their cell structures while the cells are irreversibly damaged and eventually go into necrosis (35–37). The dying tumor cells will thus maintain identifying antigens that may be used by APCs to give a strong and specific immune stimulation.

#### Type of Tumor Cell Death

Cell death through necrosis is associated with high immunogenicity, whereas cell death through apoptotic mechanisms is substantially less immunogenic (4, 20, 31, 32). Dependent on the method and the settings used, local destruction creates varying mixtures of necrosis, apoptosis and secondary necrosis, and the proportions are likely to affect the outcome. The relative impact of necrosis and apoptosis is illustrated by studies within the field of cryotherapy, which has seen conflicting results with respect to immunostimulation and immunosuppression (20). For instance, a high rate of freeze was followed by a lowered metastatic spread whereas a low rate of freeze was not. The explanation was that the extent of necrosis increases with a high rate of freeze and that the extent of apoptosis increases with a low rate of freeze (120). It has also been shown that the anti-tumor immune response is decreased when cryoablation is extensive and this has been attributed to the relatively large amount of apoptosis that is seen in this situation (20). In contrast, the feedback imILT treatment results in a low temperature peripheral zone that contains cells that maintain their cell structures while the cells are irreversibly damaged and eventually go into necrosis (35–37).

#### Tissue Blood Flow

Adequate exposure of tumor antigens and trafficking of immune cells depend on preserved tissue perfusion. The relevant area for targeting is the tumor periphery, which contains tumor cells that are viable and perfused with blood. Since most LTDs kill tumor cells together with endothelial cells, the challenge is to preserve at least some of the blood and lymph flow close to the peripheral zone of treated tumor.

Activation and function of T cells are metabolically demanding and require glucose and mitochondrial activity. Preserved blood flow helps to avoid hypoxia, which unabated leads to dysfunction of immune cells and increases immunosuppressive mechanisms in the tumor (220–223). It has been shown that mitigation of hypoxia boosts T cell activity and enhances the effect of immunotherapies such as checkpoint blockade and adoptive cell transfer (221–223).

The importance of preserved blood flow can be seen in a study that investigated the effects of using either a low-dose PDT protocol with vascular preservation, the PDT-immune stimulating approach, or a high-dose tumor ablating PDT protocol with vascular shut-down, or a combination of both. It was shown that a two-step regimen, i.e., first a low-dose immune-enhancing PDT protocol followed by a high-dose tumor-ablating PDT protocol, enhanced immunogenicity and improved tumor growth control, including lowered metastatic spread, as compared to using either regimen (97). The first step did not ablate the local tumor but preserved vascular flow, which allowed for trafficking of immune components.

Reports of vascular effects induced by irradiation have been inconsistent, but a few generalizations can be made (224). A radiation dose that exceeds 10-15 Gy/fraction gives pronounced changes of the vasculature leading to decreased blood perfusion of the tumor soon after irradiation, whereas lower doses have minimal effects (224, 225). In conventional fractionated, low dose radiotherapy the function of the tumor vasculature seems to be unchanged, or increase, during the early phase of treatment and decrease towards the end of therapy. The situation is less clear in high-dose hypofractionated radiotherapy such as stereotactic body radiotherapy or stereotactic radiosurgery, but it appears that doses higher than 10 Gy/fraction induce severe vascular damage. Most experimental studies have used high-dose single or hypofractionated radiotherapy.

Most hyperthermic treatments use temperatures above 60°C, which cause coagulative necrosis and destroy tumor antigens as well as the microcirculation in and around the tumor. The situation is different with imILT where temperature is controlled at 46°C 3 mm outside the tumor border  (37, 63). At this temperature level the microcirculation is reduced but not abolished, which allows for antigen exposure and trafficking of immune cells (226, 227).

#### Radicality

It has been known for decades that advanced tumor burden is associated with a suppressed anti-tumor immune response (40). It has been shown that T_reg_ cells disproportionally accumulate in tumors as they increase in size or enter an advanced disease stage (228–232), that the response to anti-CTLA-4 therapy is reduced at increased tumor burdens (233, 234) and that overall survival following anti-PD-1 treatment in advanced melanoma varies negatively with baseline tumor size (235). It would thus be optimal to perform local treatment with radical intent. However, most LTDs pay a price for this goal since they kill cells by rapid coagulative necrosis, which leads to destroyed tumor antigens, destruction of tumor-resident immune and endothelial cells and absence of tissue perfusion at the tumor border, which in turn limits the access for immunoactive cells and agents.

Several reports, using different LTDs, have indicated that non-radical treatments may induce immunologic systemic effects that are stronger than those obtained with radical treatments. Non-radical tumor cell kill, followed by radical treatment, was more immunogenic and gave better control of tumor growth than immediate complete destruction, for instance by RFA-induced hyperthermia or by PDT (47, 97). In a study examining cryotherapy together with LPS, it was found that killing about 70% was more efficient than killing about 90% with regard to the abscopal effect (130). Intentionally subradical (<10% of tumor volume) HIFU together with anti-CTLA-4 and anti-PD-L1 resulted in an impressive abscopal effect (114). Several radiotherapy studies have shown that a low dose, given as a single treatment or in hypofractionated regimens, gives a stronger abscopal effect than a single large dose when radiotherapy is combined with an immunomodulator (161, 201, 236, 237). In a radiotherapy only study, it was shown that treatment of 20% of the tumor volume had a greater abscopal effect than treating the whole tumor (166). Similar results have been reported in patients with bulky lung tumors in whom partial radiotherapy with sparing of the vascularized tumor periphery led to improved local control and abscopal effects, effects that were not seen when conventional, whole-tumor fractionated radiotherapy was used (203).

If the local treatment is not completely radical, it is important to try to avoid ischemic preconditioning since ischemia/reperfusion is a strong stimulus for growth of remaining tumor (238, 239).

In radiotherapy treatments, one explanation for the superior immunogenicity of low dose, as compared to high dose, may be that a high dose degrades tumor DNA which lowers production of activated DCs (237). Another explanation, and which applies to all types of local treatment, is that subradical treatment preserves lymph and blood perfusion in some parts of the tumor.

Since elimination of the treated tumor is a desirable goal, it should be pointed out that low level temperature hyperthermia offers the possibility to perform radical treatment together with preservation of tissue perfusion (226, 227). Intentionally non-radical imILT produced systemic effects but radical treatment had a superior systemic immunologic effect (78). Thus, immune enhancing and abscopal effects do not need to be incompatible with radical treatment.

### Consequences of Local Tumor Destruction Combined With Immunotherapies

The consequences of combining LTD with immunotherapy may be studied either as the ability of the LTD to boost the action of immunotherapy or as the ability of immunotherapy to boost the action of LTD. Both approaches were investigated in the majority of the preclinical studies with only a few being restricted to investigate if immunotherapy can increase the action of a certain LTD (158, 167, 175, 189). In the clinical reports, the absence of a proper control group makes it is difficult to draw unequivocal conclusions about the value of LTD, regardless of the immunotherapeutic agents used and the timing of the LTD, in combination treatments.

In the preclinical studies, the two most frequently used approaches were combinations with checkpoint blockade (n=46) and various methods to increase the participation and effect of DCs (n=28). RFA and cryotherapy were relatively often combined with DC injection or agents that improve the functions of DCs. Radiotherapy was most often combined with checkpoint blockade. Treatment with any type of LTD in combination with immunotherapy produced abscopal effects in all studies except for two studies using radiotherapy, one combined with IL-2 and the other with anti-PD-1 as the immunotherapeutic agent (156, 191).

Adding an immunotherapeutic agent increased tissue levels of CD8 at all studied sites, including treated and untreated tumor, regardless of the tissue level obtained by the LTD. This was consistent for all types of LTDs and immunomodulators. T_reg_ levels were relatively modestly affected, they were most often unchanged and sometimes lowered. The same pattern was seen for MDSCs although data are relatively limited. DC levels showed the expected rise in combination with DCs and DC stimulators. PD-L1 levels were not markedly influenced by checkpoint blockade.

#### Actions and Timing of Various Immunotherapies

In the reviewed studies, efforts to improve the immunologic effects of LTD included agents that a) enhance proliferation and function of DCs, b) stimulate proliferation, activation and efficacy of effector T cells, and c) inhibit factors that suppress the immune response.

##### Engagement of DCs

Apart from being triggered by the LTD, DC activity can be increased by injection of DCs into the tumor. It can also be promoted by DC stimulators (such as Flt3, OK-432, GM-CSF) and by modifying the influence of tumor-associated antigens by TLR agonists (such as CpG, CpG-ODN, imiquimod, BCG, BCG-CWS) and agonists of costimulatory receptors (anti-CD40).

With few exceptions, DCs and DC stimulators were administered intra- or peritumorally. They were given concurrently with, or 1-2 days after, the local treatment and often continued for one or several days.

This timing is consistent with the knowledge that immature and inactivated DCs are better than mature DCs to take up new antigens and avoids the risk that the local treatment kills DCs administered and/or activated before local treatment. A study performed by Silvestrini et al, however, suggested that giving activators of DCs before LTD may be preferable in some instances. They showed that intratumoral CpG and intraperitoneal anti-PD-1 before, and concurrent with, HIFU produced an abscopal effect that was not seen when only concurrent administration was used. They used magnetic resonance-guided focused ultrasound ablation to achieve non-radical thermal ablation which they argued increases the released amount of undestroyed antigens for presentation (112). Their results suggest that timing of DC combination therapy should take the type of LTD and immunomodulation and, maybe in particular, the extent of cell damage into consideration.

#### T Cell Proliferation and Activation

Besides proliferation and activation of DCs, means to increase the number and efficacy of effector cells included agents like CPBs, anti-OX40 and anti-CD137 in the reviewed reports. These agents were administered systemically in all but one study that used intratumoral injection of anti-PD-1 and anti-CD137 (193). The common regimen has been to start treatment with these agents at the same day as, or a few days after, local treatment and then to continue for a few days. However, timing has not received a lot of attention and the optimal timing of the various LTDs and immunotherapeutic agents remains to be established.

With regard to anti-CTLA-4, it appears reasonable that it should be given before LTD in order to start the proliferation of T cells in preparation for the response of tumor-specific T cells that will be induced by the LTD. Young et al. found that injection of anti-CTLA-4 before irradiation was more efficient than administration after irradiation, whereas anti-OX40 antibody gave an optimal effect when administered one day after irradiation (175).

Agents that increase the efficacy of effector cells, such as anti-PD-(L)1 and the agonistic stimulatory antibody anti-CD137, have usually been given concurrently with LTD and continued for some time to maintain effector T cell activity and prevent exhaustion. Starting anti-PD-(L)1 therapy at the time of LDT treatment assumes that it acts at the effector stage of pre-existing naïve T cells and targets the induced increase of PD-1 on T cells after their activation. It is also consistent with the finding that CPBs have been shown to be effective especially in a pre-existing, or created, inflammatory microenvironment together with increases of PD-L1 and T_reg_ cells (24, 217, 240). It may, however, not be the optimal timing in all situations. As described above, it has been shown that anti-PD-1 together with CpG was more efficient when it was started before than concurrent with thermal therapy (112). At any rate, a checkpoint blocker should probably not be given late after completion of fractionated radiotherapy since it has been described that anti-PD-L1 given concurrently prolonged survival, whereas it did not when it was started 7 days after ending radiotherapy (241).

IL-2 stimulates the proliferation and differentiation of T cells to cytotoxic, helper and regulatory T cells and promotes T cell differentiation into memory cells (242). IL-2 and IL-2 producing agents have been given locally and systemically and concurrent with or after LTD treatment.

##### Inhibition of Immune Suppressors

Untreated tumors grow because the factors that cause immune suppression overpower those that promote an effective immune response. Examples of therapeutic agents, other than CPBs, that decrease suppressor mechanisms already present in the TME are IDO inhibitors, TGF-β inhibitor or anti-TGF-β, anti-VEGF, anti-GITR and cyclophosphamide. These agents have usually been given before and concurrent with, and often also after, local treatment (169, 172, 180, 184, 189, 198, 200). Low dose cyclophosphamide lowers T_reg_ levels and has been shown to lead to rejection immunity when administered before PDT and cryotherapy (99, 100, 127).

When an LTD creates a T cell-inflamed TME, the inflammatory changes may be associated with activation of negative pathways that restrain the desired effect, which may account, at least partly, for the infrequent abscopal effects when an LTD is used alone. Thus, an immunotherapeutic agent that counteracts both pre-existing and LTD-induced immune suppressor activity may tip the net balance in favor of anti-tumor immune activity. A typical example is irradiation that produces immunogenicity-favoring factors but also elevations of immunosuppressive factors such as PD-L1, T_reg_ cells and TGF-β. This increase in immunosuppressive factors was counteracted with checkpoint or TGFβ blockade, contributing to an abscopal response following radiotherapy (**Table 4**).

## Conclusions

Local destruction techniques can induce or enhance changes that contribute to anti-tumor immune activity and sometimes lead to abscopal effects. In addition to the preexisting immunogenicity in the tumor, the quality and strength of the immunologic response depends on factors such as antigen exposure, type of cell death, the degree and nature of the induced T cell-inflammation in the tumor and the blood flow and perfusion in the peripheral parts of the tumor. It seems that the best results were obtained when the released antigens were not destroyed, when tumor cell death was necrotic and when tumor tissue perfusion was at least partially preserved, which should facilitate antigen presentation and immune cell trafficking and decrease the influence of hypoxia with consequent immune cell dysfunction (**Figure 1**). Treatment performed in a way that respects these requirements is likely to improve outcome when local tumor destruction is used.

An important determinant for the outcome of an LTD is the immunologic changes that it generates in the TME; ideally anti-tumor immunologic changes should be stronger than the associated immunosuppressive changes. As judged by the systemic effect, this was not usually the case for the various LTDs with the exception of low level hyperthermia (**Table 5**). In combination therapies, the type and proportion of tumor killing versus tumor-promoting immunologic changes in the TME influences the choice and effect of the immunotherapeutic agent. For example, if the LTD produces immunosuppressive changes that hamper a concomitant immune stimulation, adding an immunotherapeutic agent that deals with the immunosuppressive factor can lead to an abscopal effect.

Further knowledge of specific tissue changes produced by LTDs and immunotherapies should lead to combination protocols with improved outcomes. The observation that controlled low level hyperthermia, such as imILT and magnetic thermotherapy, regularly produces systemic effects on its own indicates that it leads to changes in the TME, where the resulting anti-tumor immunologic actions are stronger than the immune suppressive changes. It would thus be interesting to further explore the tissue changes produced with this method. Other areas for possible improvement are the timing and route of administration of the immunotherapeutic agents. These aspects have been studied rather infrequently and further studies are wanted to explore how and when different regimens can improve outcome.

The “best” LTD is yet to be defined and may differ between different histologic types of tumors and locations, cold and hot tumors and the degree of preexisting immunogenicity. At any rate, controlled low level hyperthermia, such as imILT and magnetic thermotherapy, is a good candidate, considering that it is especially prone to create a response that favors abscopal immune activity on its own.

The reviewed studies have shown that local tumor destruction in combination with immunotherapy can elicit a strong anti-tumor immune activity that results in abscopal effects. It is likely that future studies on combinatorial strategies will translate into improved outcome for many patients with advanced disease. Another advantage is that combination therapy has the potential to lower cost, dosage, and the risk of severe side-effects using immunotherapy alone.

## Author Contributions

The author confirms being the sole contributor of this work and has approved it for publication.

## Conflict of Interest

The author is co-founder and shareholder of Clinical Laserthermia Systems AB, a company that holds granted patents for the imILT method.

## Footnotes

1 Cancer immunotherapy is usually defined as a therapy that uses cellular and/or humoral elements of the immune system to treat cancer. This definition is used in this paper.
